# Growth and nutrient removal efficiency of duckweed (*lemna minor*) from synthetic and dumpsite leachate under artificial and natural conditions

**DOI:** 10.1371/journal.pone.0221755

**Published:** 2019-08-27

**Authors:** Jamshaid Iqbal, Atif Javed, Muhammad Anwar Baig

**Affiliations:** 1 Department of Environment and Energy Management, College of Business Management, Institute of Business Management (IoBM), Karachi, Sindh, Pakistan; 2 Department of Environmental Sciences, University of Okara, Okara, Punjab, Pakistan; 3 Institute of Environmental Sciences and Engineering, School of Civil and Environmental Engineering, National University of Sciences and Technology, Islamabad, Pakistan; Universiti Teknologi Petronas, MALAYSIA

## Abstract

Sustainable management of leachate produced from the dumpsite is one of the major concerns in developing countries Aquatic plants such as duckweed have the potential to remove pollutants from wastewater which can also be cost-effective and feasible options for leachate treatment. Therefore, the objective of our present study was to examine the growth and nutrient removal efficiency of duckweed (*Lemna minor)* on leachate. Three tests were performed each by growing *lemna minor* on synthetic leachate under controlled conditions and on dumpsite leachate under natural conditions. During each test, duckweed was grown in 300 ml plastic containers with a surface area of 25.8 cm^2^. About 60 mg of fresh mass of duckweed was grown on 250 ml leachate at an internal depth of 9.5 cm. Results revealed that, in comparison to synthetic leachate, duckweed removed Chemical Oxygen Demand (COD), nitrogen (N), and phosphorous (P) more efficiently from dumpsite leachate under natural climatic conditions. However, the amounts of N and P absorbed into duckweed body mass were about 16% and 35% respectively more at synthetic leachate under controlled conditions. Maximum growth rate of duckweed (7.03 g m^-2^ day^-1^) was also observed for synthetic leachate in comparison to the growth rate of 4.87 g m^-2^ day^-1^ at dumpsite leachate. Results of this study provide a useful interpretation of duckweed growth and nutrient removal dynamics from leachate under natural and laboratory conditions.

## Introduction

The absence of standard landfill sites in developing countries has given rise to the formation of open waste dumpsites which produce relatively large amounts of leachate [1]. Leachate is a type of concentrated wastewater produced at open dumpsites by percolation of rainwater through solid waste layers [2]. Composition of typical leachate is highly variable containing large amounts of pollutants and nutrients such as organic matter, ammonia-nitrogen, heavy metals, and chlorinated organic and inorganic salts [3, 4]. Pollution caused by leachate is a potential threat to the environment and human health [5]. Unattended leachate is representing a potential environmental risk to the surface as well as ground water quality [6]. There are many studies on the effect of leachate pollution on human health, flora, fauna and ecosystems [7].

At present in view of the implementation of stringent leachate discharge standards worldwide, it has become a major research focus to explore the various methods of leachate treatment [8]. A wide range of physical, chemical, biological and combination of two or more methods of leachate treatments are being practiced worldwide [3, 9]. The potential method for leachate treatment is determined by many factors such as, current waste disposal practices, geographical location of landfill/dumpsites, local weather pattern, leachate composition and economic concerns of leachate treatment [10].

Use of aquatic plants such as duckweed, water hyacinth and water lettuce etc. has been recognized and getting more attention recently in wastewater treatment [11]. Aquatic plants also offer an alternate technology of converting wastewater nutrients into potentially useful forms in addition to the treatment [12]. Duckweed is amongst the promising aquatic plants having enormous capacity to treat eutrophicated wastewaters. Wastewater treatment by duckweed is owed to its ability to accumulate large amounts of nutrients and minerals into its body mass and show high growth rates under worse environmental conditions [13,14].

Duckweed is a small floating macrophyte belonging to family *Lemnaceae* of monocotyledonous plants. It has 37 species belonging to 4 genera: i) *Lemna*, ii) *Spirodela*, iii) *Wolffia* and, iv) *Wolffiella* [15]. It is a simple plant having no stem or leaves. Major part of the plant comprises a thallus called "frond" which is generally composed of chlorenchymatous cells having air pockets called aerenchyma due to which duckweed floats on water. Duckweed may have no root or one or more simple roots. Roots are photosynthetically active having chloroplast in it. Roots of the duckweed plant help in nutrient uptake from water and stabilizes the plant [16].

*Lemna minor*, belonging to the genus *Lemna* is the most widely spread species of duckweed which is extensively studied in wastewater treatment mainly due to its fast growth and high nutrient removal efficiency [17]. Under favorable climatic conditions and nutrient balance in growth media, *Lemna minor* can double its biomass within two days [18]. Cheng et.al reported a growth rate of *L*. *minor* close to 29 g m^-2^ day^-1^ in high strength swine wastewater while the total Kjeldahl Nitrogen (TKN) and Total Phosphorous (TP) absorbed by duckweed were 90% and 88.6%, respectively [12].

Assimilation of nitrogen by duckweed fronds and roots appears to be the primary mechanism of nitrogen fixation in plant. However, some portion of nitrogen is also absorbed into duckweed biomass through associated N fixing cyanobacteria and algae grown in duckweed ponds [19]. Nitrate and ammonium are the main forms of available nitrogen for duckweed however, the absorption of ammonium is 3 to 11 times greater than nitrates. Nitrogen is fixed as protein in duckweed biomass [20]. Various studies report a variable amount of nitrogen absorbed by duckweed. Zhang et al. reported that in wastewater with initial nitrogen concentration of 12 mg N L^-1^ duckweed consumed nitrogen at the rate of 446 mg m^-2^ day^-1^ [21]. Another study reported the nitrogen absorption rate of 547±136 mg N m^-2^ d^-1^ by duckweed [22].

Unlike other vascular plants, *Lemna minor* absorbs large amount of phosphorous into its body mass [23]. However, compared to nitrogen, the phosphorous requirement of duckweed is very small for optimum plant growth [24]. Phosphate (PO_4_^-3^) is the preferred form of phosphorous uptake by the duckweed. Phosphorous makes up to 0.03 to 2.8% of a typical duckweed dry mass whereas the nitrogen content is about 0.8 to 7.8% [25]. Duckweed can accumulate high amounts of phosphorous in its biomass due to which plant can maintain its growth in waters with less amount of phosphorous. When duckweed dies, stored P in plant biomass is readily available in the water [26]. Literature shows that *Lemna minor* has varying capacity of P uptake under different environmental conditions. Duckweed shows optimum growth at phosphorus concentration of 4 and 22 mg P L^-1^ of growth media [24]. Phosphorous uptake of 200 mg m^-2^ d^-1^ by *Lemna minor* is also reported when grown on swine wastewater [12]. Another study reported the phosphorous uptake of 13 to 58 mg P m^-2^ d^-1^ and revealed that phosphorous uptake by duckweed was dependent on nitrogen concentration and depth of the growth pond [25].

Protein contents of a typical duckweed may be as high as up to 45% of the total dry mass of plants. Due to high protein content, the harvested duckweed is a potential food source for human and animal feeds [27].

Based on its wastewater treatment potential, it is hypothesized that *Lemna minor* can also be used as a cost effective and technically feasible option for leachate treatment. Therefore, present study was designed with the objective to investigate the growth of duckweed and its efficiency to remove COD and nutrients (N&P) from synthetic and dumpsite leachate. Attempts at searching for literature reveals that currently a very small amount of research has been conducted on the use of duckweed for leachate treatment. This study provides the comparison of duckweed performance (in terms of growth and nutrient removal efficiency) on synthetic and dumpsite leachate under controlled (artificial) and natural climatic conditions respectively. So far, no such comparison is available in literature however very few isolated research studies have been conducted using either natural or artificial duckweed-leachate systems. Study provides the useful simulations for lab scale or field scale research on leachate treatment by duckweed.

## Materials and methods

**Fig 1:**
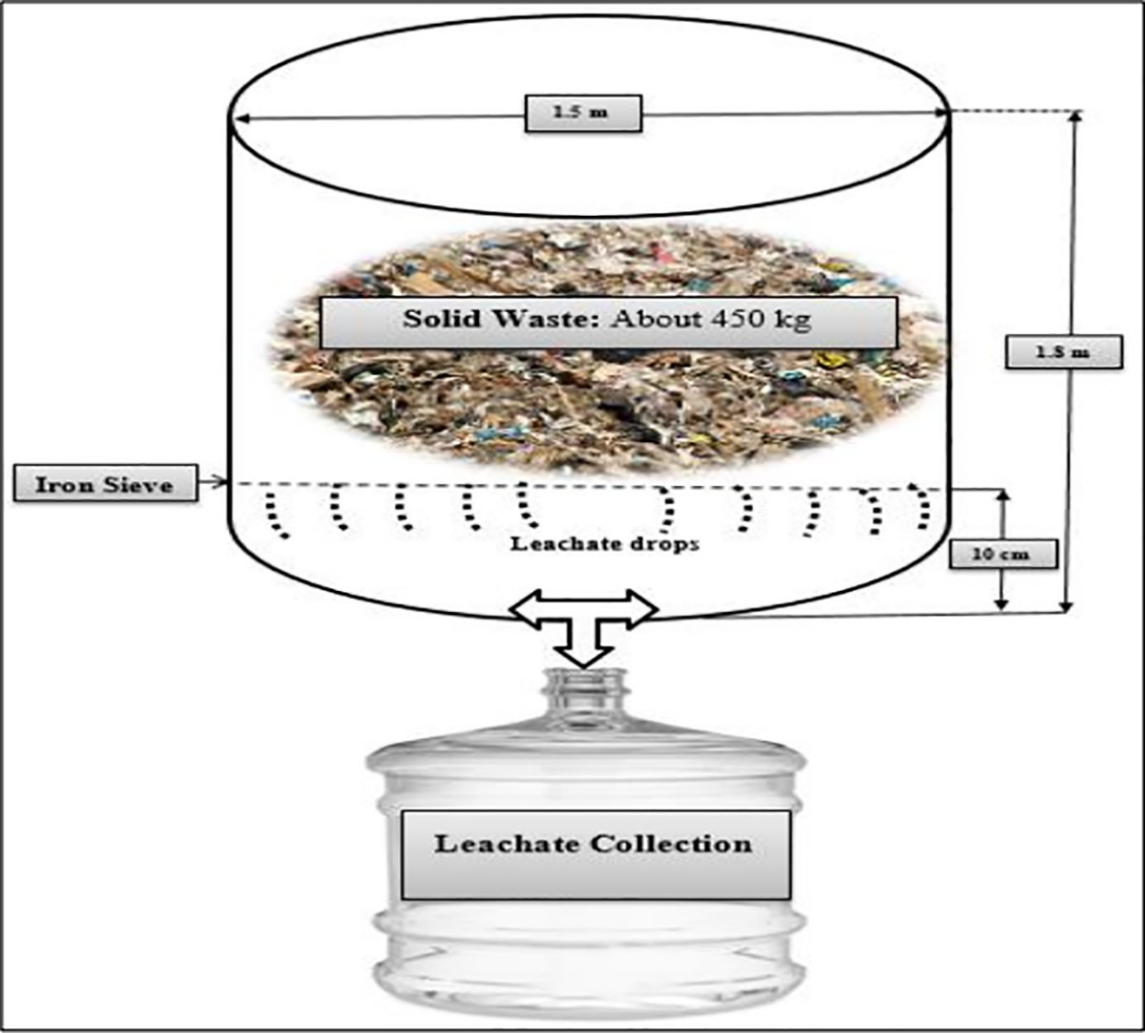
Schematic of leachate production setup.

Dumpsite leachate used in this study was prepared by processing the mixed solid waste collected from various residential, commercial and industrial dumpsites in Islamabad, Pakistan. About 100 to 120 kg of well decomposed solid waste was collected from each dumpsite. Waste was collected from pre-determined lowest points at depths of 0.5 m to 1.5 m [28]. Collected wastes were mixed in plastic tank having an internal diameter of about 1.5 m and a height of about 1.8 m. A sieve (pore size 1mm) was fixed at an internal height of 10 cm of the plastic tank. Fig 1 shows the schematic setup used for leachate production.

Synthetic leachate with COD about 1527±2.42 mgL^-1^ (approximately equal to the COD of dumpsite leachate) was prepared by adding the measured quantities of NaNO_3_, K_2_HPO_3_, KHCO_3_, K_2_CO_3_, NaHCO_3_, MgCl_2_.6H_2_O, MgSO_4_.7H_2_O, CaCl_2_ and glucose powder in distilled water. In view of the complex chemical composition of dumpsite leachate, it was difficult to prepare synthetic leachate of exactly similar composition. However, after repeated measurements and hit and trial analysis synthetic leachate with desired COD, and nutrient contents was prepared. Table 1 represents the nitrogen and phosphorous contents and COD of synthetic leachate. Because leachate composition shows high temporal variability [29], therefore an initial analysis of both types of leachate were made soon after the preparation and the analyzed leachate was immediately used for duckweed growth.

**Table 1:** Initial nutritive composition and COD (mean ± SD) of leachate used as media for duckweed growth.

Leachate Type	Nutrients concentration (mg L^-1^)				COD (mg L^-1^)
	TKN	NH_4_-N	TP	o-PO_4_^3-^-P	
**Dumpsite**	49.88±0.26	26.43±0.15	37.13±0.21	14.08±0.14	1527±2.42
**Synthetic**	46.56±0.24	24.32±0.15	42.65±0.22	16.02±0.16	1571±2.49

A mixed culture of duckweed was collected from wastewater treatment pond located in National University of Sciences and Technology (NUST), Islamabad, Pakistan. *Lemna minor* plants were isolated from the mixed duckweed culture and used for this study after acclimatization for about seven days.

Three separate tests (each comprising two parts) were performed during the month of June-July by growing duckweed on dumpsite leachate (Part 01) under natural conditions and on synthetic leachate (Part 02) under the similar artificial conditions. During each test, duckweed was grown in 300 mL plastic containers with a surface area of 25.8 cm^2^. About 60 mg of fresh mass of duckweed was grown on 250 mL leachate at an internal depth of 9.5 cm. During each part of the three tests, nine (09) containers were used in triplicate including six duckweed containers and three controls without duckweed.

For part 01 of each test, duckweed containers were placed within a meshed iron rack under natural climatic conditions whereas; for part 02, containers were placed within the growth chamber under similar controlled conditions. In the growth chamber, the required light intensity was adjusted with the help of fluorescent lamps whereas, the required day lengths were adjusted with the help of auto shut down system of fluorescent lights. Temperature adjustments within the growth chamber were made with the help of a temperature gauge fitted with the chamber. Throughout the experiments, the pH of the leachate was maintained at about 7± 0.6. using 1M solution of Hydrochloric Acid (HCl) and Sodium Hydroxide (NaOH).

Data related to ambient air temperature and day lengths as shown in Table 2 was retrieved from the website of Pakistan Metrological Department, whereas the solar radiation data was obtained from the web site of LEO Corporation, Pakistan.

**Table 2:** Weather conditions during experiments on duckweed growth on leachate.

Test Performed	Ambient temperature (^o^C)	Solar intensity (kWh m^-2^ day^-1^)	Day length (Hours)
Test 1	38.3 ± 1.0	4.3 ± 0.6	13.4 ± 0.4
Test 2	38.9 ± 0.8	4.5 ± 0.5	13.6 ± 0.4
Test 3	38.5± 1.1	4.4 ± 1.3	14.1 ± 0.7
**Average**	**38.6 ± 0.9**	**4.4 ± 0.8**	**13.7 ± 0.5**

Each test was performed for 10 days during which samples from the leachate and control containers were analyzed for TKN, ammonium nitrogen (NH_4_^+^-N), TP, ortho phosphate (o-PO_4_^-3^-P) and COD by removing three containers at the start and end of the test. Duckweed plants were oven dried at 70 ^0^C until it had a constant weight. Dried mass of duckweed was ground with the help of a mortar and pestle and then a plant extract was prepared for analysis of TKN and TP contents. Furthermore, a chemical analysis was performed using the standard methods of American Public Health Association [30]. The details of material used, and experimental techniques adopted during study are given in Table 3.

**Table 3:** Analytical instruments and apparatus used during the study.

S. No	Parameters	Analytical Instrument	Model
1.	Chemical Oxygen Demand (COD)	COD Reactor	Velp ECO 25
2.	Total Kjeldahl Nitrogen (TKN)	Semi-Automatic Kjeldahl Distillation System	KDN
3.	Ammonium nitrogen (NH_4_^+^-N)	UV Visible Spectrophotometer & Portable Spectrophotometer	PG-Motel T 60 Hitachi U2800
4.	Ortho-Phosphate-Phosphorous (o-PO_4_^-3^-P)	UV Visible Spectrophotometer Portable Spectrophotometer	Hitachi U2800
5.	Total Phosphorous (TP)	UV Visible Spectrophotometer	Hitachi U2800 PG-Motel T 60
6.	pH	pH meter	Hanna HI 8520 Eutech pH 700 WTW 720
7.	Duckweed Mass	Analytical Balance	Adam AAA 160 LE Adventure AR 3130 Phoenix, BTG-303
8.	Duckweed Drying	Oven	WTC Blinder LDO-030 N
9.	Duckweed growth under controlled conditions	Growth Chamber	Chasewood, Environmental USA

All experiments during this study were conducted within the premises of Institute of Environmental Sciences and Engineering (IESE), National University of Sciences and Technology, Islamabad, Pakistan where I am pursuing my doctoral degree (33° 38′ 41″ N, 72° 59′ 22″ E). Experimental site is owned by the IESE, NUST where no permits are required to conduct the research work for IESE students. Furthermore, it is to note that no endangered or protected species or locations were involved during this research study.

All treatments were performed in triplicate. Data collected on all parameters was analyzed statistically using Fisher’s analysis of variance (ANOVA) techniques under completely randomized design (CRD). Statistical analysis was performed using Statistix-8.1 and MS excel software.

## Results and discussion

Table 1 depicts that the nutritive composition and COD level of synthetic and dumpsite leachate has no significant difference. Therefore, for further data analysis and results interpretation, initial composition of both types of leachates is assumed to be identical. Table 2 shows that during each test there exists a very small difference in natural weather conditions. This situation was also helpful for maintaining the artificial weather conditions in the growth chamber where no significant variations of conditions was required throughout the experimental period.

Table 4 provides a comparison of duckweed growth rates at dumpsite and synthetic leachate indicating that during each test, duckweed exhibited the better growth in synthetic leachate than that of in dumpsite leachate. In synthetic leachate a maximum growth rate of 7.03 ± 1.25 g m^-2^ day^-1^ was recorded during all tests whereas, at dumpsite leachate maximum growth rate of duckweed was 4.87 g m^-2^ day^-1^. *Lemna minor* has variable growth rates under varying climatic conditions. Seasonal growth of duckweed ranges from 3 to 9.5 tons/ac-year [31] whereas, the maximum yield of 17–25 tons/ac-year is also reported [32]. In dumpsite leachate growth rates of *Lemna minor* ranging from 4.3 to 6.4 g m^-2^ day ^-1^ have also been reported [33]. Similar growth rates of duckweed (3.2 to 5.7 g m^-2^ day ^-1^) were also reported by another study conducted by growing duckweed on a dumpsite leachate under varying electrical conductivities of leachate [34]. In dumpsite leachate under the natural conditions, large amount of nutrients is removed by the other factors than absorption into duckweed biomass which results in retarded growth of duckweed plants [33]. This might be the reason of high growth rate of duckweed at synthetic leachate. Significant amounts of N and P may be removed through ammonia volatilization, nitrification and denitrification and microbial assimilation in addition to the duckweed absorption in natural leachate-duckweed systems [35].

**Table 4:** Comparison of duckweed growth rates on synthetic and dumpsite leachate during three tests.

Test Conducted	Leachate Type	Growth rate (g m^-2^ day^-1^)
Test 1	Dumpsite	4.06 ± 1.18
	Synthetic	6.84 ± 2.13
Test 2	Dumpsite	4.87 ± 1.62
	Synthetic	7.03 ± 1.25
Test 3	Dumpsite	3.89 ± 0.78
	Synthetic	6.77 ± 0.93

A comparison of COD and nutrients removal from synthetic and dumpsite leachate is provided in Table 5 indicating that during each test, compared to synthetic leachate, duckweed removed nutrients and COD more rapidly from dumpsite leachate under natural conditions.

**Table 5:** Comparison of rates of nutrients removal and COD reduction by duckweed from synthetic and dumpsite leachate.

Test Conducted	Leachate Type	Nutrients removal rate (mg m^-2^ day^-1^)				COD (g m^-2^ day^-1^)
		TKN	NH_4_-N	TP	o-PO_4_^3-^-P	
Test 1	Synthetic	116.32±0.65	80.39±1.71	92.16±0.39	26.24±0.61	2.73±4.20
	Dumpsite	152.12±0.72	133.71±0.87	109.24±1.05	38.78±0.45	3.31±5.81
Test 2	Synthetic	123.41±1.33	82.28±1.14	96.42±1.27	26.94±1.52	2.81±3.63
	Dumpsite	157.52 ± 1.62	141.83±1.36	111.92±1.35	39.15 ±1.62	3.64±4.12
Test 3	Synthetic	126.83±1.45	86.19±1.72	101.93±1.41	28.47±1.44	2.96±2.87
	Dumpsite	159.73 ± 0.98	149.38±1.59	119.08 ±0.78	42.07 ±1.71	3.81±3.63

Duckweed absorbs variable amounts of nitrogen and phosphorous under varying conditions. Absorption of nitrogen by duckweed largely depends on the initial nitrogen concentration in growth media, Chemical Oxygen Demand (COD), hydraulic retention time and duckweed plant density [36]. Iqbal and Baig reported that during summer season *Lemna minor* removed TKN and TP from dumpsite leachate at the rates of 40 to 310 mg m^-2^ day ^-1^ and 30 to 200 mg m^-2^ day^-1^ respectively [33]. While TKN and TP removal of 152 to 175 mg m^-2^ day ^-1^ and 84 to 92 mg m^-2^ day ^-1^ respectively by *Lemna minor* from dumpsite leachate under natural conditions have also been reported [34]. The high rates of nutrients and COD removal from dumpsite leachate are attributed to the processes such as ammonia volatilization, algal and microbial assimilation, and nitrification/denitrification which are high under natural duckweed-leachate systems [35]. Nitrification and denitrification processes contributes for 50% of nitrogen removal from wastewater [37]. High rates of nitrification and denitrification processes resulted by large population of respective bacteria may remove large amounts of nitrogen from dumpsite leachate.

**Fig 2:**
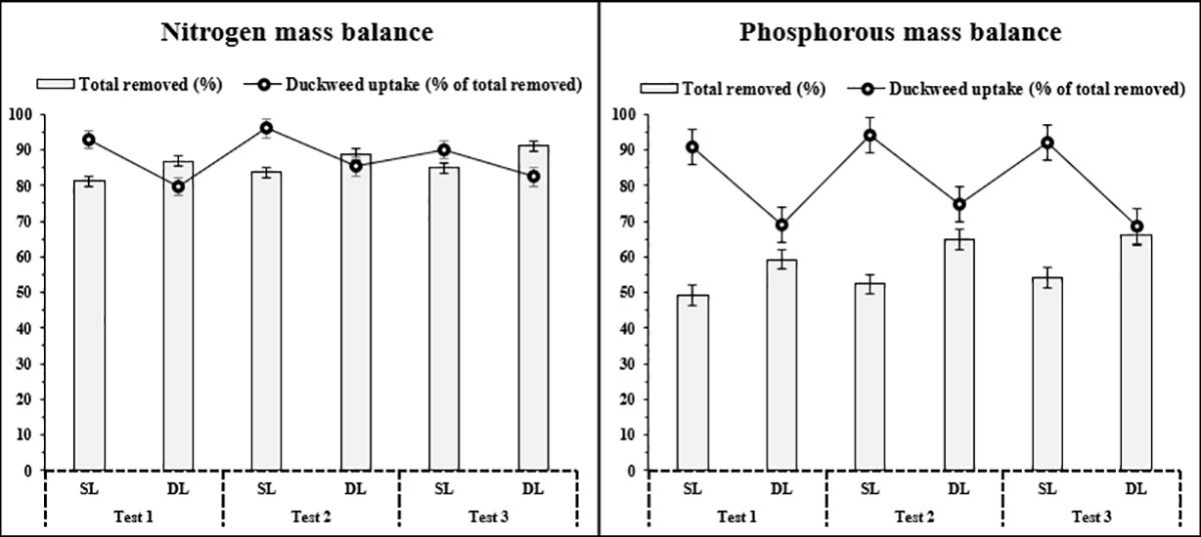
Comparison of mass balance of total nitrogen and phosphorous removal and uptake by duckweed from synthetic and dumpsite leachate.

It is evident from the comparison of mass balance that duckweed absorbed larger amounts of nitrogen and phosphorous into its biomass from synthetic leachate as compared to the absorption of these nutrients from dumpsite leachate under similar conditions (Fig 2). This is consistent with the growth of duckweed which is also high at synthetic leachate.

## Conclusions

This study provides the comparison of duckweed (*Lemna minor*) growth and its efficiency to remove COD and N & P from synthetic and dumpsite leachate. Results reveal that compared to synthetic leachate under artificial conditions, duckweed removes COD and nutrients more efficiently from dumpsite leachate. However, the amount of nitrogen and phosphorous absorbed into duckweed body mass was about 16% and 35% respectively more at synthetic leachate. The high growth rate of duckweed was also observed at synthetic leachate. In conclusion, many factors such as microbial activities, algal growth and natural decomposition also contribute to nitrogen and phosphorous removal from leachate in addition to absorption by duckweed under the natural duckweed-leachate system.

## Supporting information

S1 File Nitrogen mass balance. (XLSX)

S2 File Phosphorous mass balance. (XLSX)
